# Comparison between Three Therapeutic Options for the Treatment of Balance and Gait in Stroke: A Randomized Controlled Trial

**DOI:** 10.3390/ijerph18020426

**Published:** 2021-01-07

**Authors:** Sagrario Pérez-de la Cruz

**Affiliations:** Department of Nursing, Physiotherapy and Medicine, University of Almería, 04120 Almería, Spain; spd205@ual.es; Tel.: +34-950-214-574

**Keywords:** balance, brain injury, gait, aquatic therapy, quality of life

## Abstract

Stroke patients are more likely to be at risk of falling, which leads to limitation in their abilities to perform daily living activities and participate in society. The aim was to compare the relative effectiveness of three different treatment groups for improvements in postural control and for improvements in balance. Forty-five participants diagnosed with acquired brain injury, with over one year’s evolution, were divided into a dry land therapy group (control group), an experimental group (Ai Chi aquatic therapy), and a combined group (therapy on dry land and aquatic therapy with Ai Chi). The Berg balance scale, tandem stance, the timed up and go test, and the five times sit-to-stand test were used. After twelve weeks of treatment, the results improved significantly for the combined therapy group (*p* < 0.01), and were significantly higher compared to the dry land therapy group (*p* < 0.01). In addition, improvements were also found in the aquatic Ai Chi therapy group. In conclusion, aquatic Ai Chi and/or the combination of aquatic therapy with dry land therapy is effective for the improvement of static and dynamic balance and for enhancing functional capacity, therefore, increasing the quality of life of acquired brain injury patients.

## 1. Introduction

One of the most severe and common problems suffered by individuals with stroke is a decline in balance and an increased risk of falls. Approximately 40% of people suffer a fall within the first year [1] and one out of every three adults over the age of 65 falls each year [2,3]. Falls are associated with negative physical and psychological consequences, including injuries, loss of independence, fear of falling, and, at times, the need for institutionalization [4].

Many studies have demonstrated that physical activity prevents the risk of falls, by increasing strength in the lower limb muscles and improving gait pattern, as well as balance and coordination parameters [5,6,7,8]. A comprehensive assessment of postural control and balance (both static and dynamic) is recommended in order to design an appropriate and personalized exercise program for fall prevention. It is important to be able to identify both the disturbed systems that underlie the control of balance as well as the specific rehabilitation activities that should be used to achieve the expected results [9].

The current evidence includes several studies that support the use of aquatic therapy for the improvement of gait in adults with neurological illnesses [6,7,8], however, there are few studies focused on the reeducation of balance using different types of aquatic therapy. Furthermore, there is a lack of studies specifically on these therapies in adults with stroke.

One aquatic therapy technique that may improve the stability in standing as well as the gait pattern and balance in these patients is aquatic Ai Chi [10]. This consists of a series of aquatic exercises created by Jun Konno in Japan in 1996, based on the combination of concepts such as Tai-Chi and Qigong. During the Ai Chi sessions, an instructor verbally and visually teaches a combination of movements that involve the upper and lower limbs and the trunk in a slow and coordinated manner. The participants practice these movements while they are standing in the water at shoulder height level [10].

For this reason, and considering the evidence from Ai Chi therapy in other neurological pathologies [11,12], this study aims to evaluate the effectiveness of this therapy with patients with stroke. Therefore, the purpose of this study was to compare the effects of three treatment groups: dry land therapy, Ai Chi therapy in the water, and the combination of both therapies, over 12 weeks, considering parameters related to postural control and balance. We hypothesize that, in comparison with a group that received conventional dry land physiotherapy, the group of individuals performing aquatic exercise and the group that received combined aquatic therapy and dry land physiotherapy would display an improvement in their motor disability and balance.

## 2. Materials and Methods

### 2.1. Study Design

This study was a randomized, single-blinded controlled trial (NCT04168164). The participants were recruited from three brain injury associations and those who fulfilled the inclusion criteria were randomly assigned to three treatment groups. These were: an experimental group (AQ group), which received aquatic therapy, a control group (PT group), which received training on dry land, and a combined group (AQ + PT group), which received combined sessions of aquatic therapy and dry land therapy.

Initially, the participants were assessed at baseline, prior to beginning the intervention sessions. The tests were performed by a blind assessor, i.e., the person who performed the measurements was blinded to the study group of each subject. A simple randomization procedure was used, with unmarked envelopes and a 1 in 3 possibility of belonging to each group. They were re-evaluated at the end of the sessions and, finally, one month after finishing the treatment.

The research protocol was approved by the Bioethics Committee of the university of the main author (UALBIO2017/007). All the participants provided informed consent in writing before participating. This study was conducted in accordance with the Helsinki Declaration.

### 2.2. Participants

**Figure 1:**
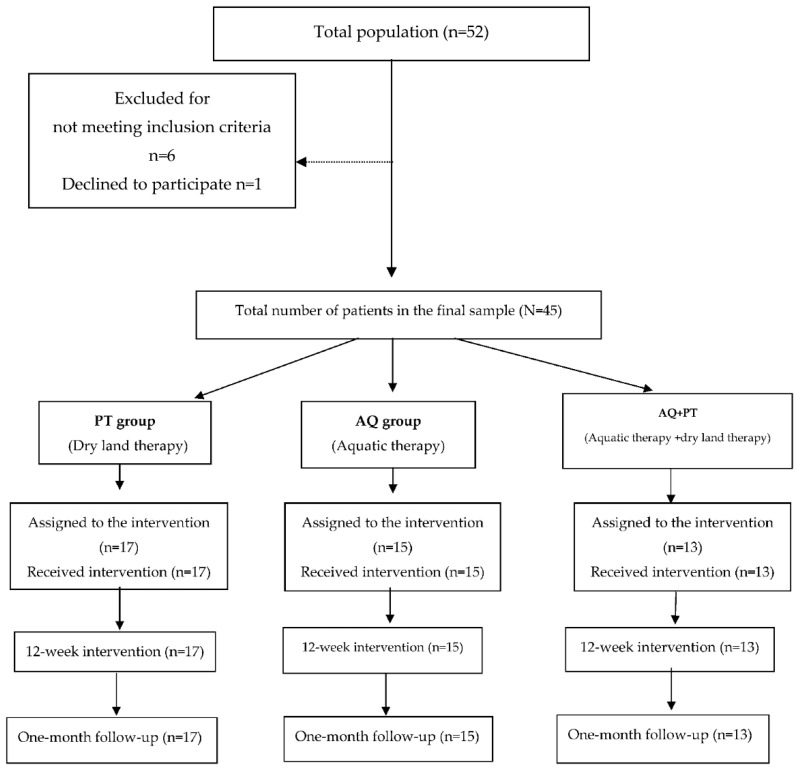
Study design flowchart.

In total, 45 patients participated in the study, during a six-month recruitment period. According to the random allocation, 13 patients formed the combined group (AQ + PT group), 17 were part of the PT group (dry land therapy), whereas 15 were assigned to the AQ group (aquatic therapy). There were no significant differences in the demographic and clinical variables among groups. Figure 1 displays the selection and randomization process of patients assigned to the intervention groups.

The admission criteria for the study were: (1) a diagnosis of stroke, with over one year’s evolution, (2) the ability to walk independently over a distance of more than 10 m, with or without an assistive device, (3) scoring >24 on the Mini-Mental State Examination, and (4) the absence or mild presence of spasticity, with a score on the modified Ashworth scale less or equal to 2 in the paretic lower limb. Those with any contraindication for working in water were excluded, including cardiovascular and/or respiratory problems, a previous history of neurological pathologies, the presence of coadjuvants to the stroke, or any musculoskeletal disorder that may affect their participation in the study.

### 2.3. Outcome Measures

The participants were assessed with the following evaluations before initiating therapy, after completing therapy, and one month after finishing the last session:

The Berg balance scale, calculated as the sum of scores of 14 different functional tasks and balance tasks [13,14]. The scores on the Berg balance scale were classified as follows: high risk of falls (0–20), moderate risk (21–40), and low risk (>41) [14]. The test consists of sitting, getting up, maintaining the standing position, and changing positions, with a total of 14 items. The total possible score is 56 points, with higher scores indicating that the person has better balance.

The tandem stance with eyes open (TSEO) test. This test measures the amount of time that the participant could maintain this position, measured for up to 30 s. Three attempts were allowed and the attempt with the best score was used [15].

The five times sit-to-stand test (FTSTS) [16] was used to assess the functional mobility. The participants were seated with their arms crossed and they were asked to stand up and sit down five times as fast as possible. 

The timed up and go test (TUG) was used to measure the time taken from sitting on a chair with the arms at rest, walking 3 m when a signal is given, walking 3 m back to the chair, and sitting [17]. 

### 2.4. Intervention

#### 2.4.1. Dry Land Therapy (PT Group)

Seventeen participants were assigned to the dry land therapy group (PT group) and they received 24 twice-weekly sessions in total, over a period of 12 weeks. These sessions consisted of group sessions of supervised training lasting 45 min each. These comprised a 10 min warm-up that included exercises for gait and trunk mobility and exercises involving the upper and lower limbs. The central part of the sessions consisted of 30–40 min of strength training and aerobic exercises, both individually and in groups. Each session was performed with a specific intensity goal, in order to end with a cooling down period. The session comprised 20 min of functional exercises based on activities of daily living, balance exercises, facial muscle exercises, proprioceptive exercises, muscle relaxation, and stretching. During each session, emphasis was placed on training the trunk and lower limbs with the goal of improving the overall posture. The training predominantly incorporated standing postures and a floor and balance series. Subsequently, more advanced postures and transitions were progressively incorporated into the training. 

#### 2.4.2. Aquatic Ai Chi (AQ Group)

Fifteen participants were assigned to the aquatic therapy group (AQ group) and they received 24 twice-weekly sessions in total, during the same period as the PT group (12 weeks). These 24 sessions consisted of group sessions lasting 45 min. The intervention was performed by an expert physiotherapist trained in clinical Ai Chi who was not involved in the study.

The sessions took place in a pool measuring 20 m × 6 m, at a depth of 110 cm. The water temperature was 30 °C (with variations of less than 0.5 °C) and the room temperature was 27.5 °C (with variations of less than 1 °C). The dimensions of the pool were ideally suited for collective treatment.

The sessions were designed with a gradual increase in difficulty. Initially, a recreational warm-up activity was performed, followed by 30 min dedicated to practicing the Ai Chi program. At the end of the session, there was a calming down activity. The exercises were performed in a consecutive fashion in a specific order, until all 19 movements were completed.

#### 2.4.3. Combined Group (AQ + PT Group)

This group was those who received joint sessions of aquatic therapy and dry land therapy. Thirteen patients received alternate sessions of therapy on dry land (Monday and Wednesday) and aquatic therapy with Ai Chi (Tuesday and Thursday), in the same conditions as the participants of the PT group and of the AQ group (24 twice-weekly sessions in total of 45 min). 

### 2.5. Statistical Analysis

Descriptive statistics were reported as the mean and standard deviation (SD). The Shapiro–Wilk test was used to assess the normality of data. For each outcome variable considered, the effect of the three different rehabilitation protocols was assessed by a two-factor analysis of variance: the first factor was the treatment group and the second factor was time.

To determine whether one treatment improved the scores of the study variables compared with the other, two-factor ANOVA tests were performed with general linear model (GLM) repeated measures. 

A *p* value of <0.05 was considered statistically significant. When multiple comparisons were carried out, the Bonferroni correction was applied. All analyses were carried out using the SSPS-23 statistical package (IBM Corp, Armonk, NY, USA).

## 3. Results

The final study sample comprised 45 participants, of which 48.9% were females and 51.1% were males, aged between 24 and 71 years, with an average of 52.7 years (SD = 13.3). According to the random assignment of the AQ group, 13 patients formed the AQ + PT (aquatic therapy + dry land therapy) group, 17 formed the PT (dry land therapy) group, and 15 formed the AQ (aquatic therapy) group. There were no significant differences in the demographic and clinical variables between groups (Table 1).

Table 2 shows the results obtained in each of the assessments used to evaluate the three groups at three different time points (before initiating therapy, after completing therapy, and one month after finishing the last session).

The analysis of the results obtained in the outcome measures, for the main effect of time, shows significant differences in the evolution of patients independent of the treatment group. Nonetheless, there was a significant effect for the interaction of group and time, which indicates that the time effect influenced the patients differently, depending on the group. 

After twelve weeks of treatment, the scores for the Berg balance scale, the FTSTS, the tandem stance test, and the TUG test improved significantly for the AQ + PT group (*p* < 0.01). These improvements were significantly higher for the combined therapy group (AQ + PT group) compared to the PT group (*p* < 0.01), although improvements were also found in the assessments performed in the AQ group. 

Likewise, the benefits obtained in the AQ + PT group were maintained over time (one month after completing the therapeutic intervention) and also displayed an improvement in scores, whereas for the group that only received aquatic therapy, although an improvement was found upon completing the program, the results of the Berg balance scale and the tandem stance test were not significant. 

**Figure 2:**
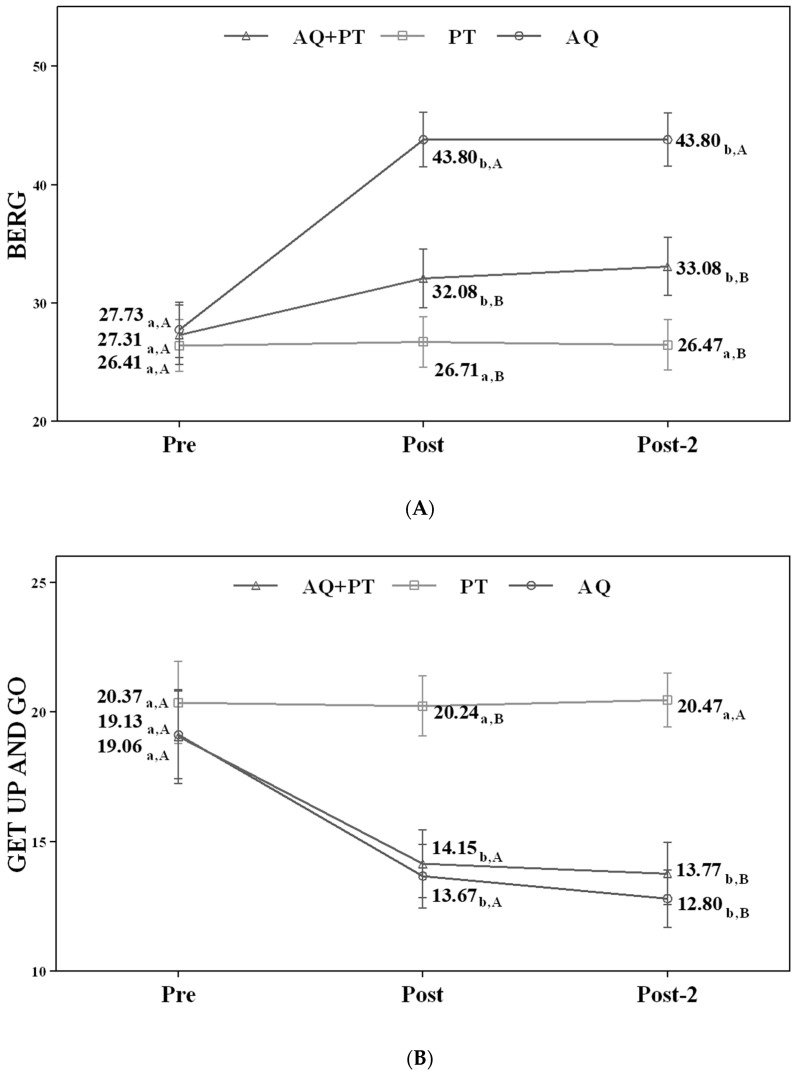

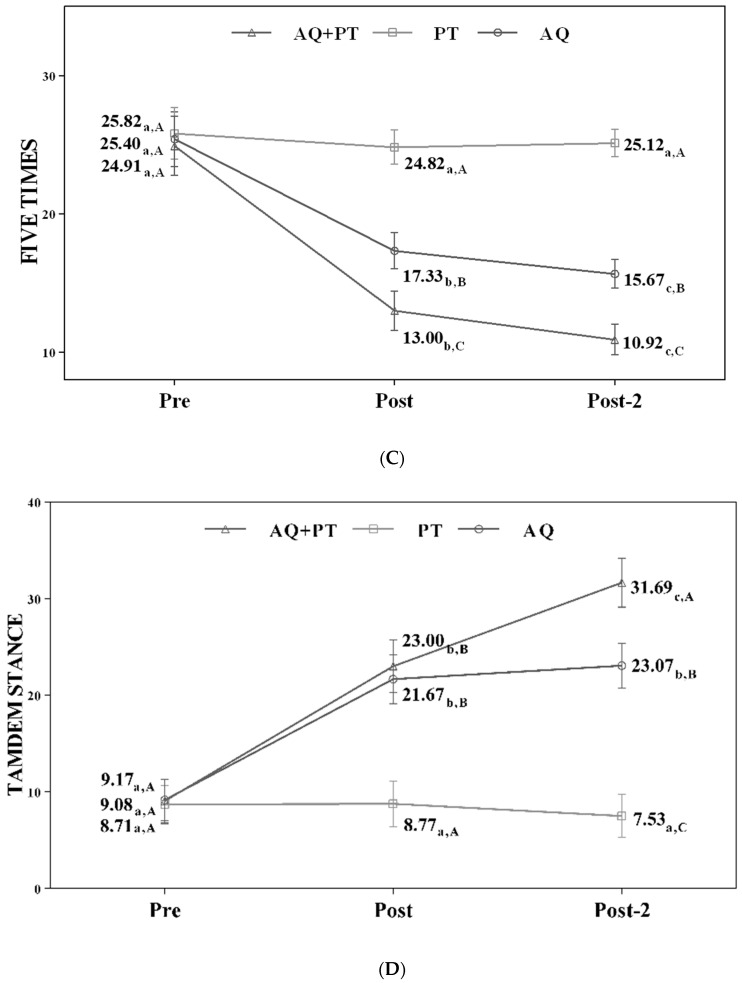
(**A**) Values of Berg balance scale; (**B**) values of the timed up and go test; (**C**) values of the five times sit-to-stand test; (**D**) values of the tandem stance test.

Figure 2 displays changes observed in the values of each of the scales used in the assessment of the participating groups. 

a–b—In the same group, different lower case letters indicate statistically significant differences between time moments (Bonferroni correction). 

A–B—At the same point in time, different capital letters indicate statistically significant differences between the groups (Bonferroni correction).

## 4. Discussion

The popularity of aquatic physiotherapy has increased among professionals, patients, and researchers due to the benefits provided by water [18]. As the body is immersed in water, it is unloaded, which enables the joints to be mobilized by the patient with reduced effort. Additionally, aquatic rehabilitation provides motor and sensory stimuli that may induce neuronal plasticity [19], improving the motor function and the static and dynamic balance of people who have suffered acquired brain injury [20,21,22]. 

Among the literature found by the author on the effectiveness of hydrotherapy for the treatment of stroke, several studies may be highlighted; some of these are based on the Halliwick method [23,24,25,26], others use Ai Chi [27,28,29], and others involve aquatic therapy, in general [30,31].

In the case of Halliwick therapy, the reports revealed improvements both at the level of balance (static and dynamic) as well as for body symmetry [23]. A study by Dong Koog et al. [24] compared the benefits obtained via conventional neurological treatment and a combined Halliwick and Ai Chi intervention. After eight weeks of treatment, improved scores were found for balance (according to the Berg scale) and strength for the hemiparetic leg in the group treated with hydrotherapy. Furthermore, according to the authors, a water temperature of between 33 °C and 34 °C may produce hyperemia in the skin, dilate the blood vessels, and promote muscle relaxation, decreasing the sensation of pain and even spasticity. This study, which included a longer treatment period, albeit with a lower water temperature, achieved similar results in terms of balance improvements.

Martínez-Gramage et al. [25] applied a combined program of physical exercise (therapy on dry land) and aquatic therapy for three months, reporting that an improvement was found in joint mobility, although statistically significant changes were not observed regarding spasticity. This research is based on the mobility of the ankle and the hypertonicity of the soleus muscle. In our case (three months of intervention), we also obtained improvements in the joint ranges (this was observed in the time taken to walk a distance and fluidity of the gait movement, timed up and go test). Therefore, aquatic therapy is also beneficial for treating spasticity, which, in these patients, constitutes a significant limitation for their movements and motor control responses.

This change could be due to the individual characteristics of each patient and the stage of the illness. It would be interesting to perform future studies with larger samples in order to determine the effectiveness of this therapy. Lastly, the effects of the aquatic method on mobility are assessed in patients with stroke in the post-acute phase [26]. After comparison with the control group (conventional therapy), significant improvements were found on the Berg scale and the functional capacity of gait in the aquatic group. These improvements coincide with those found in the present study, confirming the viability and usefulness of employing aquatic therapy for the treatment of patients of this type.

Literature on aquatic therapy with the Ai Chi method confirms the usefulness and benefits of this therapy in different pathologies of neurological origin, where the problem of balance and gait is a problem with a high index of disability. Studies [27,28,29] have demonstrated that balance training in water is safe, representing a modality that helps assist sessions on dry land and is well accepted by participants and, furthermore, it helps improve the balance of neurological patients. One of the causes that can influence the acceptance of aquatic therapy, and one of its benefits for patients with balance problems, is that they are able to overcome their fear of falling, entailing both physical and psychological benefits. By combining physical activity and a safe environment, the results obtained indicate that combined therapy or even aquatic therapy itself is a therapeutic option worthy of consideration when proposing action protocols.

Furthermore, Zhu et al. [30] performed a comparison between the results obtained with the dry land therapy vs. water therapy to improve gait and balance after a stroke. After four weeks of treatment, benefits were obtained in both groups, although in two of the tests performed (functional reach test and the 2 min walk test) the scores were significantly higher in the patients treated with hydrotherapy. Furthermore, thanks to the water temperature, there was an improvement in the blood circulation, as well as muscle relaxation, and the sensitivity to pain decreased and the motor function was optimized. In contrast, our study has highlighted that therapy on dry land has not been very beneficial in the treatment of these problems. This may be due to the use of protocols that targeted overall mobility, and were not very specific to balance problems, and a possible perception of lack of safety among patients regarding falls.

Other authors have complemented the use of aquatic therapy with other alternatives, such as dual-task training in water. Yang et al. [32] have demonstrated that dual-task training positively affects the static balance index of patients with acquired cerebral injury. Recent reports show that dual-task training in water has a positive effect on balance in patients with stroke [33,34]. Dual-task training in water uses water resistance to improve muscle strength, thereby improving the balance capacity [34,35]. The floatability in water is also compatible with bodyweight, increasing the stability of the posture and improving balance. 

Based on the literature and the results obtained in this study, aquatic dual-task training seems to be useful for improving balance in patients with stroke. Exercise in this environment also improves the strength of the lower limb muscles, via the participation of different muscle activities, which leads to important improvements in the gait capacity [36,37]. Therefore, aquatic dual-task training has a positive effect on balance and gait, by activating the sensory afferent pathways in these patients.

In this study, no adverse events were reported during the aquatic training provided to patients with stroke, confirming that aquatic Ai Chi is a safe and stimulating activity for patients with acquired brain injury.

### Study Limitations

This study has several limitations, namely the small sample size. A larger, randomized controlled clinical trial is needed in order to validate and confirm the reported benefits of this study. Furthermore, the therapist was not blinded to the group of exercises, which, although unavoidable, may lead to bias.

## 5. Conclusions

In conclusion, aquatic therapy with Ai Chi and/or the combination of aquatic therapy with dry land treatment as part of a training intervention in patients with stroke seem to be effective to improve the static and dynamic balance, the functional capacity, and, therefore, the quality of life of patients with brain damage. These improvements may persist for at least one month after completion of the program. These findings support the inclusion of aquatic therapy in exercise programs targeted at the functional rehabilitation of individuals with stroke.

## Figures and Tables

**Table 1:** Demographic and clinical characteristics of the study participants.

	Participants			*p*
	AQ + PT Group (n = 13)	PT Group (n = 17)	AQ Group (n = 15)	
Age (years)	61.4 (13.9)	62.7 (13.4)	63.8 (13.6)	0.896
Sex				0.672
Females	5 (38.5)	9 (52.9)	8 (53.3)	
Males	8 (61.5)	8 (47.1)	7 (46.7)	
Clinical history				0.754
No	10 (76.9)	11 (64.7)	10 (66.7)	
Yes	3 (23.1)	6 (35.3)	5 (33.3)	
Surgical interventions				0.254
No	3 (23.1)	9 (52.9)	6 (40)	
Yes	10 (76.9)	8 (47.1)	9 (60)	
Medication				0.059
No	4 (30.8)	0 (0)	3 (20)	
Yes	9 (69.2)	17 (100)	12 (80)	
BMI (kg/m^2^)	25.4 (2.7)	24.6 (2.3)	25.1 (3.3)	0.708
Time since the lesion (years)	5.6 (3.1)	5.2 (2.7)	5.1 (4.2)	0.113

AQ + PT group: aquatic therapy and dry land therapy group. PT group: dry land therapy. AQ group: aquatic therapy group.

**Table 2:** Mean values (SD) of the clinical evaluations and intra-subject GLM effects.

Variables	Mean Values (SD)			Intra-Subject Effects ^†^	
	Pre	Post	Post-2 (1 Month)	Time	Treatment Time
				F (d.f.); *p*-Value (eta^2^)	F (d.f.); *p*-Value (eta^2^)
Berg balance scale (points)				F(1.0; 43.1) = 66.42; *p* < 0.001 (0.613)	F(2.1; 43.1) = 30.08; *p* < 0.001 (0.589)
AQ + PT group (13)	27.31 (11.7)	32.08 (10.4)	33.08 (10.4)		
PT group (17)	26.41 (6.5)	26.71 (6.3)	26.47 (6.1)		
AQ group (15)	27.73 (9.0)	43.80 (9.9)	43.80 (9.9)		
Timed up and go test (seconds)				F(1.2; 48.4) = 47.29; *p* < 0.001 (0.530)	F(2.3; 48.4) = 5.91; *p* = 0.004 (0.219)
AQ + PT group (13)	19.06 (5.3)	14.15 (3.8)	13.77 (3.8)		
PT group (17)	20.37 (6.1)	20.24 (4.9)	20.47 (4.9)		
AQ group (15)	19.13 (7.9)	13.67 (5.2)	12.80 (3.9)		
Five times sit-to-stand (seconds)				F(1.2; 49.8) = 41.85; *p* < 0.001 (0.499)	F(2.4; 49.8) = 9.06; *p* < 0.001 (0.301)
AQ + PT group (13)	24.91 (4.4)	13.00 (3.8)	11.92 (2.8)		
PT group (17)	25.82 (6.4)	24.82 (5.5)	25.12 (4.5)		
AQ group (15)	25.40 (10.7)	17.33 (5.7)	15.67 (4.3)		
Tandem stance (seconds)				F(1.6; 68.4) = 93.98; *p* < 0.001 (0.691)	F(3.3; 68.4) = 33.79; *p* < 0.001 (0.617)
AQ + PT group (13)	9.08 (8.8)	23.00 (14.4)	31.69 (14.3)		
PT group (17)	8.71 (6.8)	8.77 (6.2)	7.53 (4.4)		
AQ group (15)	9.17 (9.0)	21.67 (7.9)	23.07 (7.3)		

Eta^2^: partial eta squared (effect size). ^†^ Greenhouse–Geisser correction. GLM: generalized linear model. AQ + PT group: aquatic therapy and dry land therapy group. PT group: dry land therapy. AQ group: aquatic therapy group.

## Data Availability

Data sharing not applicable.
